# Exome-Wide Association Study Identifies East Asian-Specific Missense Variant *MTHFR* C136T Influencing Homocysteine Levels in Chinese Populations RH: ExWAS of tHCY in a Chinese Population

**DOI:** 10.3389/fgene.2021.717621

**Published:** 2021-10-11

**Authors:** Tianzi Liu, Mohetaboer Momin, Huiyue Zhou, Qiwen Zheng, Fangfang Fan, Jia Jia, Mengyuan Liu, Minghui Bao, Jianping Li, Yong Huo, Jialin Liu, Yaning Zhang, Xuemei Mao, Xiao Han, Zhiyuan Hu, Changqing Zeng, Fan Liu, Yan Zhang

**Affiliations:** ^1^ CAS Key Laboratory of Genomic and Precision Medicine, Beijing Institute of Genomics, Chinese Academy of Sciences, Beijing, China; ^2^ China National Center for Bioinformation, Beijing, China; ^3^ Department of Cardiology, Peking University First Hospital, Beijing, China; ^4^ Institute of Cardiovascular Disease, Peking University First Hospital, Beijing, China; ^5^ University of Chinese Academy of Sciences, Beijing, China; ^6^ Beijing P4 Healthcare Institute, Beijing, China; ^7^ CAS Key Laboratory of Standardization and Measurement for Nanotechnology, CAS Key Laboratory for Biomedical Effects of Nanomaterials and Nanosafety, CAS Center for Excellence in Nanoscience, National Center for Nanoscience and Technology of China, Beijing, China; ^8^ School of Nanoscience and Technology, Sino-Danish College, University of Chinese Academy of Sciences, Beijing, China

**Keywords:** homocysteine, hyperhomocysteinemia, MTHFR C677T, MTHFR C136T, coding variants, population heterogeneity

## Abstract

Plasma total homocysteine (tHCY) is a known risk factor of a wide range of complex diseases. No genome scans for tHCY have been conducted in East Asian populations. Here, we conducted an exome-wide association study (ExWAS) for tHCY in 5,175 individuals of Chinese Han origin, followed by a replication study in 668 Chinese individuals. The ExWAS identified two loci, 1p36.22 (lead single-nucleotide polymorphism (SNP) rs1801133, *MTHFR* C677T) and 16q24.3 (rs1126464, *DPEP1*), showing exome-wide significant association with tHCY (p < 5E−7); and both loci have been previously associated with tHCY in non-East Asian populations. Both SNPs were replicated in the replication study (*p* < 0.05). Conditioning on the genotype of C677T and rs1126464, we identified a novel East Asian-specific missense variant rs138189536 (C136T) of *MTHFR* (*p* = 6.53E−10), which was also significant in the replication study (*p* = 9.8E−3). The C136T and C677T variants affect tHCY in a compound heterozygote manner, where compound heterozygote and homozygote genotype carriers had on average 43.4% increased tHCY than had other genotypes. The frequency of the homozygote C677T genotype showed an inverse-U-shaped geospatial pattern globally with a pronounced frequency in northern China, which coincided with the high prevalence of hyperhomocysteinemia (HHCY) in northern China. A logistic regression model of HHCY status considering sex, age, and the genotypes of the three identified variants reached an area under the receiver operating characteristic curve (AUC) value of 0.74 in an independent validation cohort. These genetic observations provide new insights into the presence of multiple causal mutations at the *MTHFR* locus, highlight the role of genetics in HHCY epidemiology among different populations, and provide candidate loci for future functional studies.

## Introduction

Plasma total homocysteine (tHCY) is a known risk factor of a wide range of complex diseases, including cardiovascular and cerebrovascular diseases (Nygard et al., 1995; Refsum et al., 1998; Ganguly and Alam, 2015), type 2 diabetes (Wijekoon et al., 2007), age-related macular degeneration (Pinna et al., 2018), chronic kidney disease (Ji et al., 2019), Alzheimer’s disease (Mansoori et al., 2012), and cancers (Hasan et al., 2019).

Plasma tHCY variation has a known genetic component with a heritability estimated ranging from 47 to 70% in different populations (Jee et al., 2002; Kullo et al., 2006; Bathum et al., 2007; Siva et al., 2007; Nilsson et al., 2009). To date, 12 independent genome-wide association studies (GWASs) for tHCY have been conducted, among which nine were carried out in populations of European decent (Souto et al., 2005; Hazra et al., 2009; Malarstig et al., 2009; Pare et al., 2009; Tanaka et al., 2009; Wernimont et al., 2011; van Meurs et al., 2013; Williams et al., 2014; Shane et al., 2018), two in Africans (AFRs) (Kim et al., 2016; Raffield et al., 2018) and one in Filipinos (Lange et al., 2010). These studies collectively identified several genomic loci showing genome-wide significant association with tHCY levels. The one on chromosome 1p36.22 has been most extensively reported, where a single missense variant of the Methylenetetrahydrofolate Reductase gene (*MTHFR* C677T, rs1801133) showed an extraordinarily large effect on tHCY levels (Liew and Gupta, 2015; Moll and Varga, 2015); i.e., compared with wild-type carriers, individuals with homozygote T alleles had on average 31.08% increased tHCY levels (Shane et al., 2018). MTHFR is the rate-limiting enzyme in methionine metabolism, while the 677T variant results in a thermolabile enzyme that is ∼70% less effective during the conversion of 5,10-methyltetrahydrofolate (5,10-MTHF) to 5-methyltetrahydrofolate (5-MTHF) (Liew and Gupta, 2015). Since 5-MTHF acts as a cofactor in tHCY methylation to methionine, deficiency of MTHFR eventually leads to increased tHCY levels and a higher risk of multiple diseases (Goyette et al., 1994). Besides 1p36.22, DNA variants in or close to *CBS* on 21q22.3 (Pare et al., 2009; Lange et al., 2010; van Meurs et al., 2013; Williams et al., 2014), *NOX4* on 11q14.3 (Pare et al., 2009; Lange et al., 2010), *CPS1* on 2q34 (Lange et al., 2010; van Meurs et al., 2013; Williams et al., 2014), *DPEP1* on 16q24.3 (Pare et al., 2009; Lange et al., 2010; van Meurs et al., 2013), and *CUBN* on 10p13 (Tanaka et al., 2009; van Meurs et al., 2013) were identified for association with tHCY by at least two independent GWASs, thus representing the most robust genetic findings.

China has a pronounced prevalence of hyperhomocysteinemia (HHCY), particularly in regions with low dietary folate intake (Hao et al., 2003; Yang et al., 2013; Yang et al., 2014), which also accounted for an increased risk of ischemic stroke in patients with hypertension in these regions (Clarke et al., 2002; Li et al., 2015). Surprisingly, no genome scans for tHCY have been conducted in Chinese populations nor in any East Asian populations. Therefore, the questions regarding the possibility of the presence of multiple causal mutations and East Asian (EAS)-specific alleles of tHCY, the genetic explanation for the geospatial pattern of HHCY prevalence in China, and the predictability of HHCY risk in Chinese population remained to be answered. To answer these questions, we conducted an exome-wide association study (ExWAS) of plasma tHCY level in a Chinese cohort including 5,175 individuals, followed by a replication study in 668 individuals of Chinese Han origin.

## Results

### Discovery Exome-wide Association Study of Total Homocysteine in a Chinese Cohort

The discovery stage ExWAS included a total of 5,175 living residents of Beijing of Chinese Han origin. This cohort is characterized with a mid-aged distribution (mean age 57.14 ± 8.93 years), a pronounced proportion of females (62.17%), considerable proportions of current smokers (19.74%) and alcohol users (23.59%), and a relatively small proportion of vitamin B supplementation users (8.75%). The population structure analysis using northern (CHB) and southern (CHS) Chinese from the 1000 Genomes Project (McVean et al., 2012) as reference indicated that most of our discovery individuals are more likely to be northern Chinese (Supplementary Figure S1). The tHCY level (median 11.95 μmol/L, mean 14.09 μmol/L, sd 8.64 μmol/L) was on average higher than that reported by Schurks et al. in 44,147 Europeans (detailed sample characteristics was provided in Supplementary Table S1). In this study, age, sex, smoking, drinking, creatinine, vitamin B usage, and the first two principal components were adjusted for their potential confounding effects in association analyses.

**FIGURE 1:**
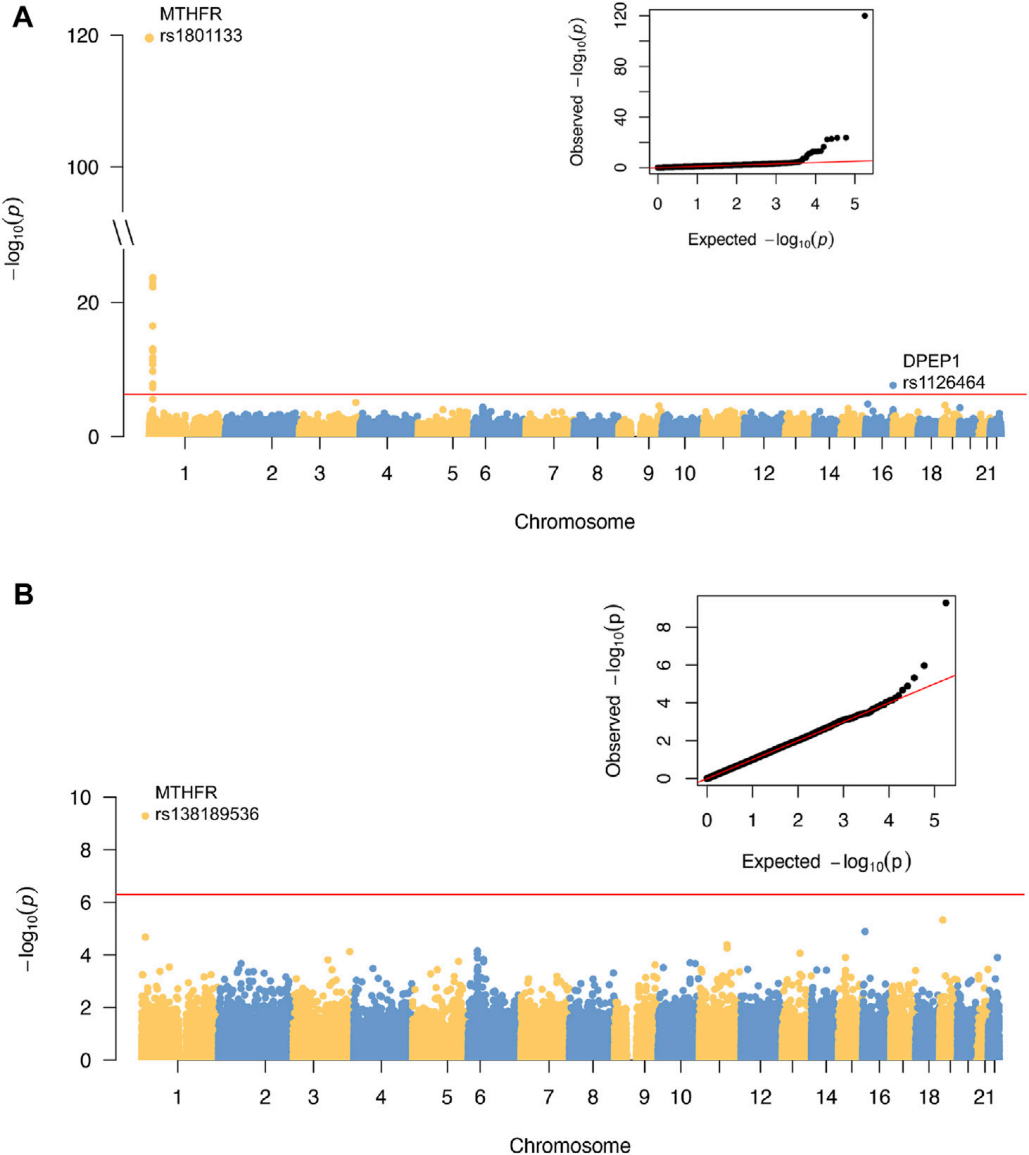
Results of ExWAS analysis and conditional analysis of tHCY in discovery cohort. **(A)** Manhattan plot and Q-Q plot of the ExWAS. **(B)** Manhattan plot and Q-Q plot of the conditional analysis. For Manhattan plots, the horizontal axes are genomic location (GRCh37.p13) of all the tested SNPs. The vertical axes are the negative log of *p*-values. Red lines indicate the threshold for exome-wide significance at 5E−7. ExWAS, exome-wide association study; tHCY, total homocysteine; SNP, single-nucleotide polymorphism.

A total of 89,131 quality-controlled, mostly coding autosomal DNA variants were tested for association with tHCY. These included those with minor allele count (MAC) >3 to detect rare coding variants with large effects. The distribution of genomic principal components (Supplementary Figure S2), the genomic inflation factor (*λ* = 1.03), and the distribution of the observed test statistics (Figure 1) did not reveal any evidence for the presence of population substructure. The ExWAS identified a total of 18 variants at two distinct loci showing exome-wide significant association with tHCY (*p* < 5.0E−7, Figure 1A; Table 1).

**TABLE 1:** Exome-wide significant variants associated with tHCY levels from ExWAS.

						Discovery (N = 5,175)			Replication (N = 668)			fEA in 1000 Genomes Project		
SNP	Gene	Chr	MBp	EA	OA	fEA	BETA	*p*	fEA	BETA	*p*	EAS	EUR	AFR
**ExWAS**														
rs4845881	*C1orf167*	1	11.83	G	A	0.19	−0.21	3.20E−17				0.27	0.31	0.52
rs4846049	*MTHFR*	1	11.85	T	G	0.15	−0.28	2.83E−24				0.23	0.32	0.53
rs2274976	*MTHFR*	1	11.85	T	C	0.06	−0.32	8.15E−14				0.12	0.03	0.02
rs1801133	*MTHFR*	1	11.86	T	C	0.58	0.45	1.26E−120	0.55	0.41	1.66E−13	0.3	0.36	0.09
rs17367504	*MTHFR*	1	11.86	G	A	0.06	−0.31	1.57E−13				0.12	0.14	0.11
rs13306561	*MTHFR*	1	11.87	G	A	0.06	−0.31	1.47E−13				0.13	0.14	0.24
rs17037425	*CLCN6*	1	11.87	A	G	0.06	−0.31	1.11E−13				0.13	0.12	0.21
rs1023252	*CLCN6*	1	11.9	T	G	0.14	−0.28	4.93E−23				0.22	0.28	0.17
rs5063	*NPPA*	1	11.91	T	C	0.06	−0.29	5.12E−12				0.12	0.03	0.06
rs198388	*NPPB*	1	11.92	T	C	0.13	−0.19	1.69E−11				0.2	0.43	0.64
rs12406089	*NPPB*	1	11.92	G	C	0.12	−0.22	1.71E−13				0.19	0.34	0.39
rs6676300	*NPPB*	1	11.93	G	A	0.14	−0.18	1.85E−10				0.21	0.37	0.63
rs11804222	*NPPB*	1	11.94	A	G	0.04	−0.29	5.01E−08				0.05	0.1	0.23
rs17037526	*NPPB*	1	11.96	T	C	0.37	−0.2	1.60E−23				0.48	0.34	0.26
rs7551175	*PLOD1*	1	12.01	A	G	0.17	−0.15	1.45E−08				0.27	0.15	0.57
rs2336384	*MFN2*	1	12.05	G	T	0.48	0.2	2.00E−24				0.37	0.34	0.31
rs2295283	*MIIP*	1	12.08	G	A	0.49	−0.14	1.68E−12				0.45	0.27	0.2
rs1126464	*DPEP1*	16	89.7	C	G	0.32	−0.12	2.22E−08	0.29	−0.11	0.048	0.36	0.25	0.08
^a^ **Conditional analysis**														
rs138189536	*MTHFR*	1	11.86	T	C	0.01	0.57	6.53E−10	0.01	0.75	9.80E−03	2.00E−03	0	0

Note. All variants with p < 5E−7 in discovery ExWAS and conditional analysis, and replication results of the three lead SNPs (rs1801133, rs1126464 and rs138189536) are shown.

Allele frequencies in East Asians (EAS), Europeans (EUR), and Africans (AFR) were obtained from the 1000 Genomes Project.

EA, effect allele; OA, other allele; fEA, frequency of effect allele; ExWAS, exome-wide association study; tHCY, total homocysteine; SNP, single-nucleotide polymorphism.

a Association result after conditioning on the genotypes of rs1801133 and rs1126464.

**FIGURE 2:**
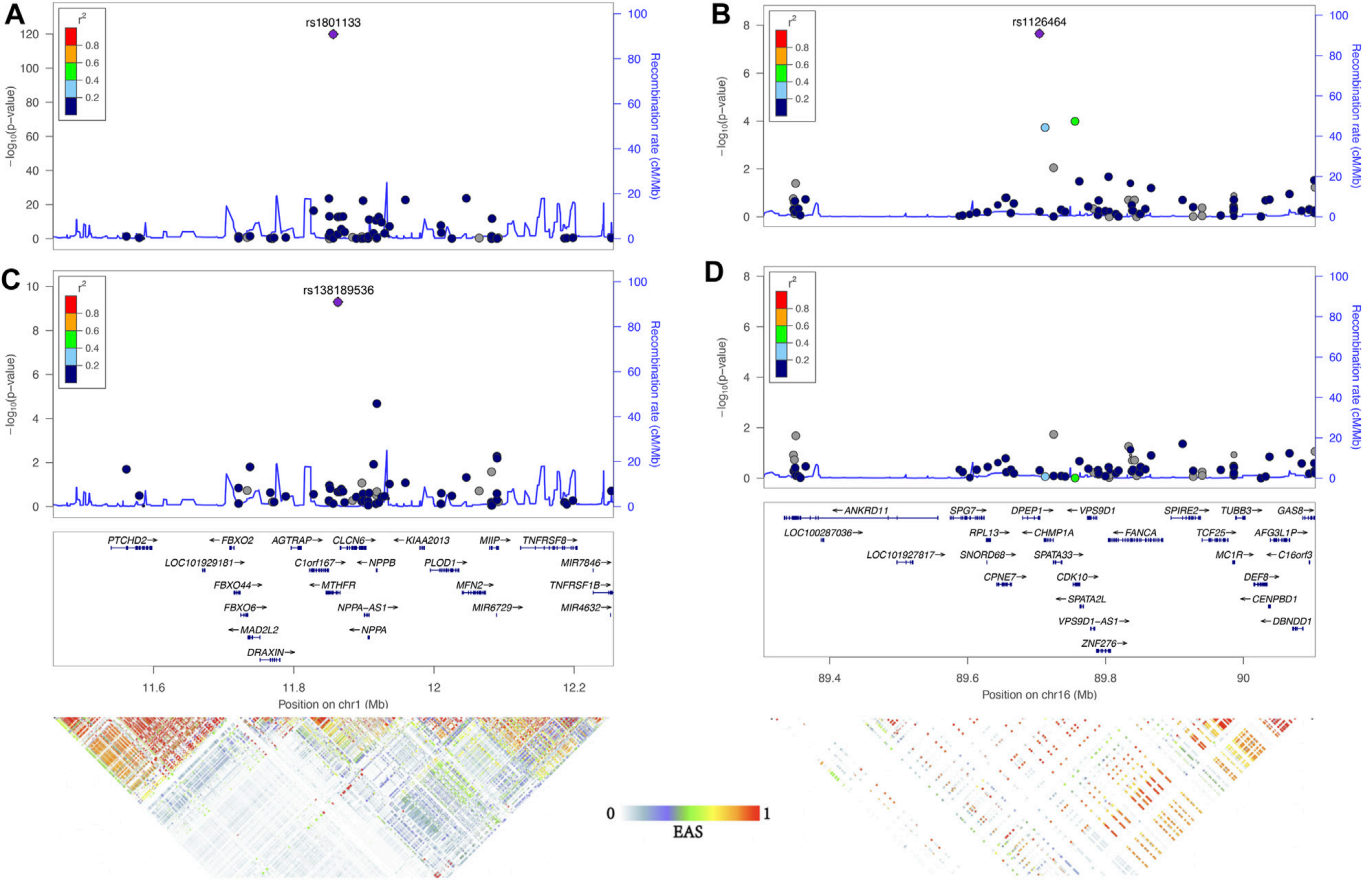
Association of two loci significantly associate with tHCY. Regional association plots were generated by Locuszoom of 1p36.22 and 16q24.3 of the results of ExWAS (**A** and **B**) and conditional analysis (**C** and **D**) using linkage disequilibrium information from the November 2014 release of the 1000 Genomes Project ASN samples. The index SNP in each region is shown as a purple diamond. Genes in the region and LD heatmap (*r*2) patterns according to the 1000 Genomes Project EAS dataset are aligned on the bottom. tHCY, total homocysteine; ExWAS, exome-wide association study; SNP, single-nucleotide polymorphism; LD, linkage disequilibrium.

**FIGURE 3:**
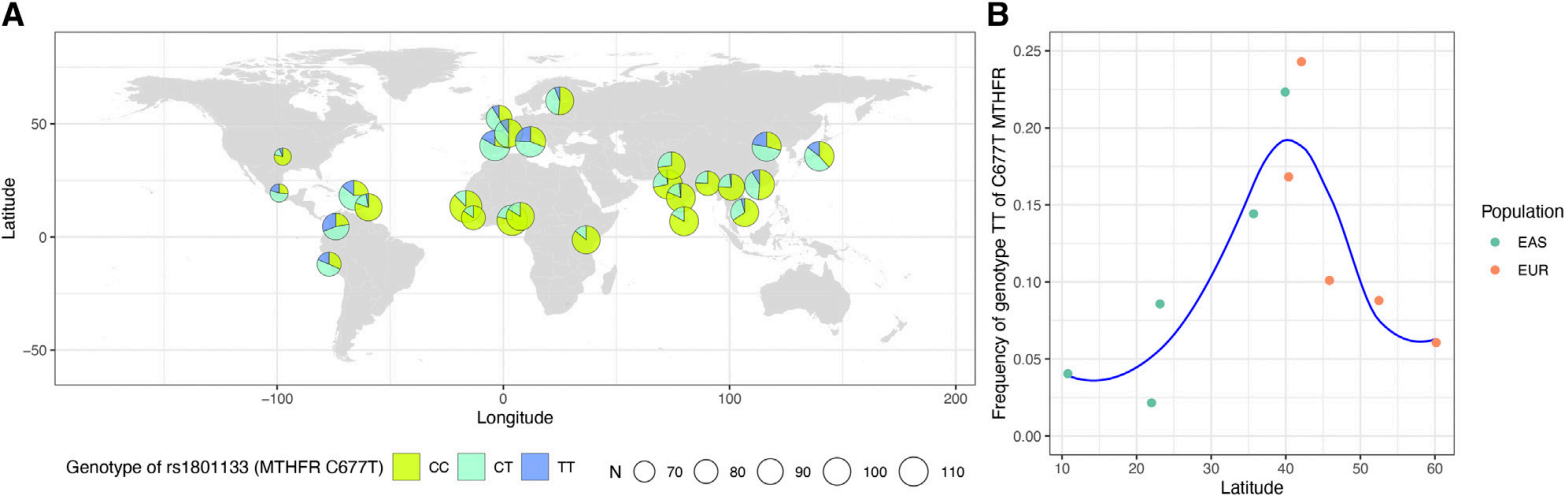
Worldwide prevalence patterns for genotypes of *MTHFR* C677T (rs1801133). **(A)** Map displaying the geospatial distribution of the prevalence patterns for genotypes of *MTHFR* C677T across the world. The map was drawn based on the genotypes of 2,504 subjects obtained from the 1000 Genomes Project datasets for this SNP. The pie denotes the sampling locations. **(B)** The frequency of TT genotype of *MTHFR* C677T based on the genotypes of East Asians and Europeans obtained from the 1000 Genomes Project datasets and the corresponding latitude of each population. SNP, single-nucleotide polymorphism.

The first locus was on chromosome 1p36.22, consisting of 17 significant variants (Figure 2A; Table 1), and harbored eight known genes: *C1orf167*, *MTHFR*, *CLCN6*, *NPPA*, *NPPB*, *PLOD1*, *MFN2*, and *MIIP*. This locus included the well-known missense variant of *MTHFR* (rs1801133, C677T, *β* = 0.45, *p* = 1.3E−120, Table 1), which was also the lead single-nucleotide polymorphism (SNP) of this locus. In our sample, 18.5% had the CC genotype of *MTHFR* C677T, 46.7% had the CT genotype, and 34.8% had the TT genotype. Individuals with the TT genotype had an on average 46.22% higher tHCY level than those with CC genotypes (17.4 vs. 11.9 μmol/L, *p* = 6.8E−60, Supplementary Table S2), explaining 6.66% of the total phenotype variance and 9.95% of the covariate-adjusted phenotype variance. In the 1000 Genomes Project data (Li et al., 2019), the geospatial distribution of the frequency of the tHCY-increasing T allele showed a latitudinal inverse-U-shaped gradient in Europe and EAS continents (Supplementary Figure S3), with the highest frequency observed around latitude of 40° north (44–46%), i.e., southern Europe (Spain and Italy) and northern China. The frequency of homozygote TT genotype showed a similar geospatial distribution with a pronounced frequency (17–24%) around latitude 40° north (Figure 3). In China, the frequency of the TT genotype showed a remarkable difference between northern China around latitude 40° (22% in CHB from the 1000 Genomes Project) and southern China around latitude 20° (9% in CHS from the 1000 Genomes Project, Figure 3A), which largely coincides with the higher HHCY prevalence in northern China.

The second locus was detected on chromosome 16q24.3 consisting of only one exome-wide significant signal, which was an exon variant of *DPEP1* (rs1126464, *β* = −0.12, *p* = 2.2E−8, Figure 2B; Table 1). rs1126464 has been previously associated with tHCY in Europeans and Filipinos (Pare et al., 2009; Lange et al., 2010; van Meurs et al., 2013) with similar allele effects as observed in our sample. This SNP was also associated with waist circumference, hypertension, and osteoarthritis in previous studies. Individuals homozygous for the major allele G (mean tHCY = 14.1 μmol/L, Supplementary Table S2) had an on average 11.02% higher tHCY level than those with CC genotypes (mean tHCY = 12.7 μmol/L, *p* = 1.5E−6, Supplementary Table S2). The frequency of the tHCY decreasing C allele also showed large differences between major populations (36% in EASs, 25% in Europeans and 8% in AFRs, Table 1; Supplementary Figure S4). Besides, according to the GTEx database (GTEx Consortium, 2013), rs1126464 is a *cis*-regulated expression quantitative trait locus (eQTL) of *CDK10*, *CHMP1A*, *FANCA*, and *VPS9D1* in multiple tissues, such as whole blood, tibial artery, skin, and thyroid (Supplementary Table S3; Supplementary Figure S5).

### Conditional Analysis of the Genotypes of Top-Associated Single-Nucleotide Polymorphisms

**FIGURE 4:**
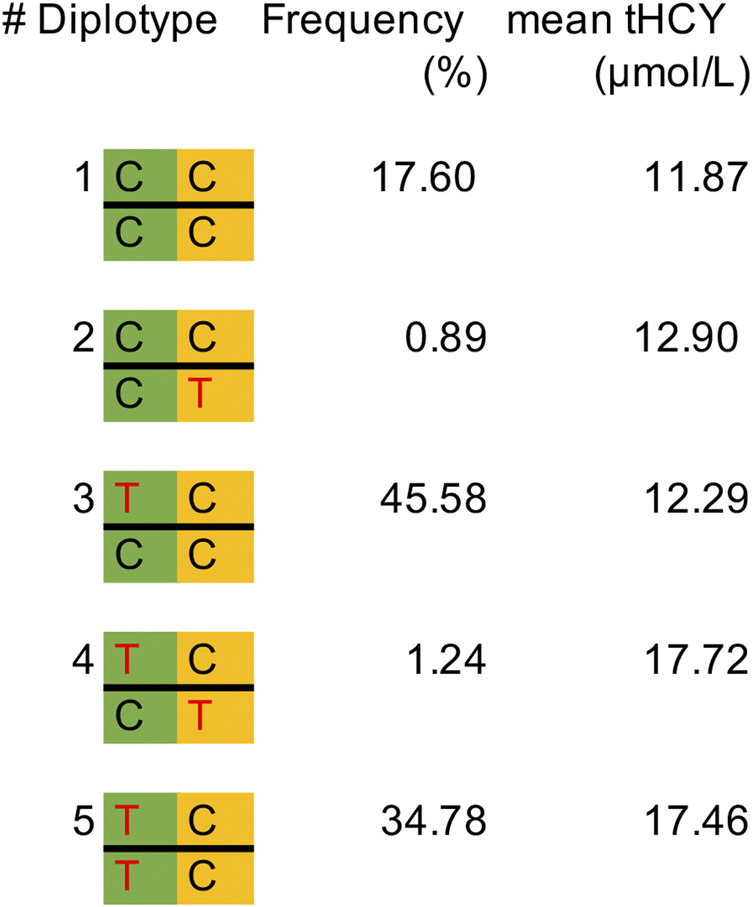
Frequency of diplotypes and the mean tHCY level in the discovery sample. *MTHFR* C677T is indicated in the green background, and *MTHFR* C136T is indicated in the orange background. Missense alleles 677T and 136T are indicated in red color. tHCY, total homocysteine.

Next, we repeated the exome scan conditioning on the genotypes of *MTHFR* C677T and *DPEP1* rs1126464, and we identified one novel rare missense variant showing exome-wide significant association with tHCY (rs138189536, *MTHFR* C136T, *p* = 2.18E−3 before conditional analysis, and *p* = 6.53E−10 after conditional analysis, Figures 1B, 2C, 2D; Table 1). This C136T variant was in weak linkage disequilibrium (LD) with the C677T variant (*r* ^2^ = 0.014). A previous enzyme kinetics experiment has shown that the arginine to tryptophan substitution at C136T led to a reduced enzymatic activity of *MTHFR*, which provided direct evidence for a functional role of this variant, consistent with our observation of the presence of multiple causal mutations at this locus (Tan et al., 2021; Weile et al., 2021). A haplotype analysis further showed that these two alleles (677T and 136T) together affect tHCY in a compound heterozygote manner (Figure 4). The compound heterozygote of 677T and 136T and the homozygote 677T carriers had on average 43.4% increased tHCY than had the wild-type and any-heterozygote carriers (17.5 vs. 12.2 μmol/L, Figure 4), and carriers of compound heterozygote of C136T and C677T had a slightly (1.5%) increased tHCY compared with homozygote 677T and 136C carriers (17.7 vs. 17.5 μmol/L, Figure 4). Note that the homozygote 136T genotype was not observed by previous studies due to its low frequency in EASs and its absence in non-Asian populations, which also explained the less significant association observed for C136T relative to C677T. The 136T allele was rare in our sample (1% in both discovery and replication samples, Table 1) as well as in the EAS sample from the 1000 Genomes Project (0.2%, Table 1). This allele is absent in non-Asian populations, which may explain the failure of previous GWASs in detecting its effect (frequency of C136T was 0 in both EUR and AFR from the 1000 Genomes Project, Table 1).

### Replication in an Independent Chinese Cohort

A replication study was conducted in a total of 668 individuals of Chinese Han origin (Supplementary Table S4). As expected, *MTHFR* C677T was again highly significantly associated with tHCY with a similar effect size as observed in the discovery ExWAS (*β* = 0.41, *p* = 1.7E−13, Table 1). The frequency of homozygote 677TT genotype in replication sample (32%) was similar as observed in the discovery sample. The 16q24.3 rs1126464 in *DPEP1* showed a nominally significant association with tHCY, and its allele effect was similar to that observed in the discovery ExWAS (*β* = −0.11, *p* = 0.048, Table 1). Conditioning on the genotype of *MTHFR* C677T and *DPEP1* rs1126464, we successfully replicated the C136T with a similar effect size as observed in the discovery ExWAS (*β* = 0.75, *p* = 9.8E−3, Table 1).

### Multivariable and Prediction Analyses

**FIGURE 5:**
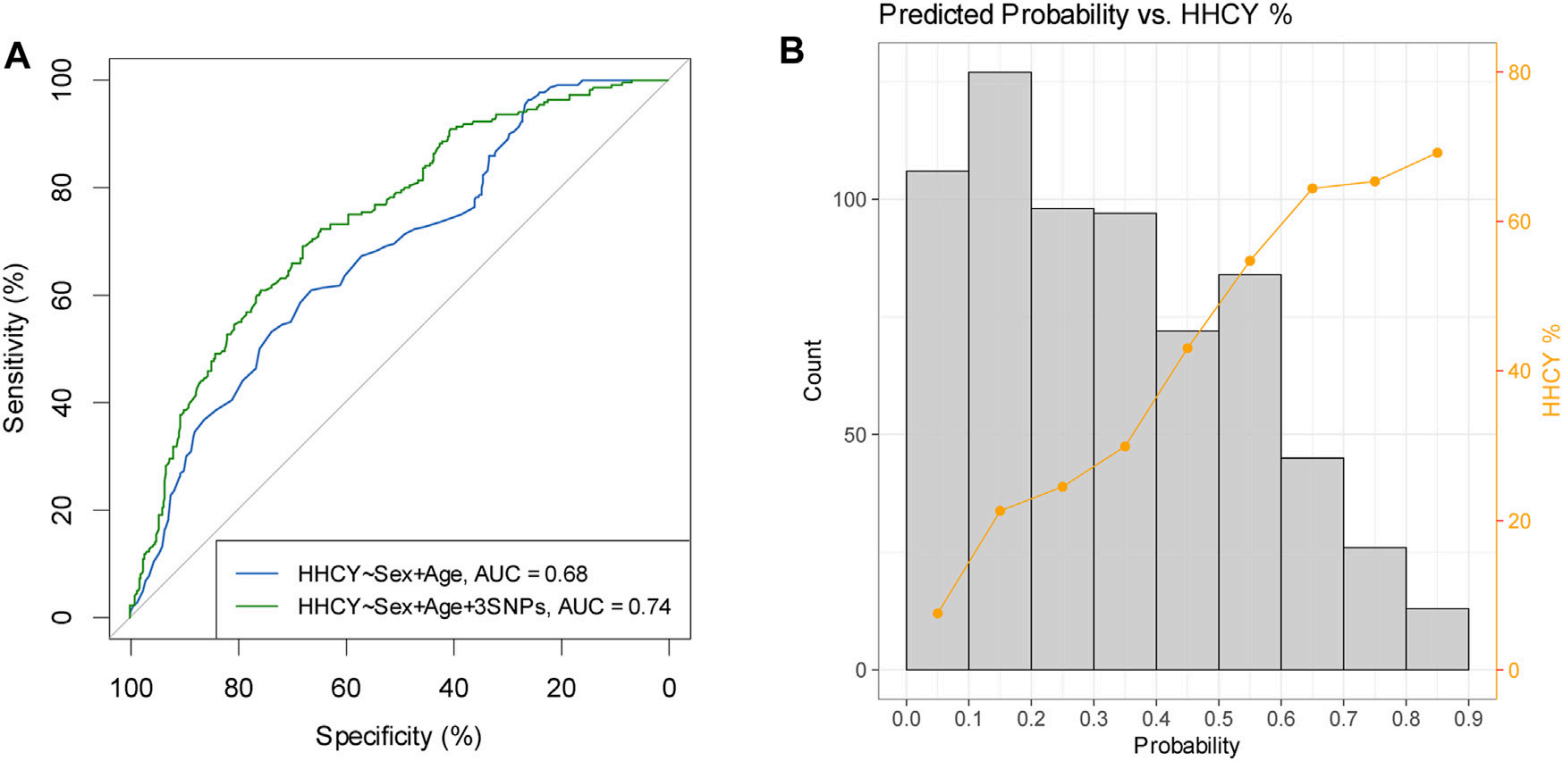
Prediction results of HHCY prediction in replication samples (N = 668). **(A)** Receiver operating characteristic curves for predicting HHCY status of prediction models considering sex and age as predictors with three identified SNPs (green line) or without three identified SNPs (blue line). **(B)** Histogram of predicted probability overplayed with percentage of HHCY in each probability bin (prediction model based on sex, age, and three identified variants as predictors). HHCY, hyperhomocysteinemia; SNP, single-nucleotide polymorphism.

A multiple regression analysis including sex, age, and the three abovementioned variants (*MTHFR* C677T, *MTHFR* C136T, and *DPEP1* rs1126464) revealed that these variables together explained 18.6% of tHCY variance in the discovery individuals (Table 2). We then constructed linear and logistic models in the discovery sample by including sex and age as predictors with or without the three identified variants and used these models to predict the tHCY levels and the HHCY status (defined as tHCY >15 μmol/L) in the replication sample. The linear model consisting of sex, age, and the three variants provided a higher accuracy in explaining tHCY levels (*R*2 = 0.14) than did the model without the three variants (*R*2 = 0.05). The logistic model consisting of sex, age, and the three variants also provided a higher prediction accuracy (area under the receiver operating characteristic (ROC) curve (AUC) = 0.74, Figure 5A) in predicting HHCY status (defined with tHCY ≥15 μmol/L) than did the model without the three variants (AUC = 0.68, Figure 5A). Although the AUC values were lower than a clinically desired level (AUC = 0.85) for diagnosis, our prediction model consisting of sex, age, and the three variants provided fairly accurate prediction results for a good proportion of individuals, that is, individuals with predicted probabilities of HHCY <0.2 or >0.8 (34.9% < 0.2 and 5.8% > 0.8, Figure 5B; also see Supplementary Table S5). In practice, our model may provide an informative test for a total of 40.7% of these individuals with predicted probabilities of HHCY <0.2 or >0.8 but less informative for the rest.

**TABLE 2:** Explained variance of tHCY by three identified SNPs.

Marker	Gene	Function	Chr	MBp	EA	OA	Beta	SE	*p*	*R* ^2^
Sex (female)							−5.79	0.22	1.66E−143	0.114
Age (years)							0.04	0.01	6.70E−04	0.115
rs1801133	*MTHFR*	CNS (C677T)	1	11.86	C	T	−3.16	0.15	3.65E−94	0.181
rs138189536	*MTHFR*	CNS (C136T)	1	11.86	T	C	3.52	0.74	2.10E−06	0.185
rs1126464	*DPEP1*	CNS (E351Q)	16	89.70	C	G	−0.51	0.16	1.41E−03	0.186

Note. *R* ^2^, accumulative *R* ^2^ while the current marker is included in a multiple regression model; CNS, coding nonsynonymous SNP; EA and OA, the effect allele and the other allele, respectively; tHCY, total homocysteine; SNP, single-nucleotide polymorphism.

## Discussion

This study represents the first genome scan of tHCY in a well-sized population of Chinese Han origin. The extraordinary large effect of *MTHFR* C677T on tHCY was confirmed in Chinese populations. The geospatial distribution of the frequency of the 677T homozygous genotype is consistent with the HHCY incidence in China, serving as a supplementary explanation for the hypothesis that folate inadequate intake accounts for the high prevalence of HHCY in northern China (Hao et al., 2003; Yang et al., 2013; Yang et al., 2014). Importantly, we detected the presence of multiple causal mutations at *MTHFR* by identifying a rare EAS-specific missense mutation (*MTHFR* C136T), which together with C677T affects tHCY in a compound heterozygote manner. This emphasized the need of sequence of the entire gene in genetic testing for HHCY. We also verified the effect of a known tHCY-associated exon variant of *DPEP1* in Chinese populations. Using these three variants, we proposed a risk assessment model of HHCY, which may be informative for approximately 40% of Chinese individuals.

We observed the strongest evidences for association between *MTHFR* C677T and tHCY in both of the discovery and replication samples. This variant alone explained approximately 10.0% of the total covariate-adjusted variance of tHCY in the discovery sample. In our study, the tHCY level of the homozygous 677T carriers was on average 50% higher than the wild-type carriers, consistent with the findings from non-East Asian populations. Besides its effect on tHCY, the 677T allele has also been reported to affect vitamin B9 (folate) and B12 concentrations. MTHFR irreversibly reduces 5,10-MTHF to 5-MTHF, and 5-MTHF was converted to the tetrahydrofolate (THF) form of folate during the conversion of HCY to methionine. Further, function studies have revealed that *MTHFR* C677T results in ∼70% decrease of enzyme activity and thereby accompanied by decreased plasma folate level and increased tHCY level (Scott and Weir, 1981; Liew and Gupta, 2015).

C136T has been reported as a missense mutation of *MTHFR*, which may increase the occurrence and recurrence of pulmonary embolism (Tan et al., 2021). To our knowledge, this is the first genome scan revealing the influence of *MTHFR* C136T on tHCY. It is noticeable that C136T is only present in EASs, highlighting the importance of well-sized association study on tHCY in Chinese. C136T and C677T together influence tHCY in a compound heterozygote manner, consistent with the role as missense variants of both C136T and C677T in reducing the activity of MTHFR enzyme, supporting the previous findings of the existence of multiple causal alleles on *MTHFR* (Burda et al., 2015). Clearly, sequencing the entire gene is preferred for genetic testing involving *MTHFR*.

HHCY is a strong risk factor of cardiovascular and cerebrovascular diseases, type 2 diabetes, and cancers (Nygard et al., 1995; Refsum et al., 1998; Schurks et al., 2008; Mansoori et al., 2012; Ganguly and Alam, 2015; Pinna et al., 2018; Hasan et al., 2019; Ji et al., 2019). The prevalence of HHCY is very high in China, particularly in northern China (Yang et al., 2014), which has been hypothesized to account for an increased risk of multiple diseases in northern China. Previous epidemiological studies have revealed that the high prevalence of HHCY was likely due to the inadequate intake of folate. In this study, we observed that the frequency of homozygous *MTHFR* 677TT genotype is as high as 0.32 to 0.35 in northern China, compared with 0.09 in southern China. This observation may serve as an alternative or supplementary explanation of the large difference of HHCY prevalence between northern and southern China. The observation that 677T being a “bad” allele and at the same time a major allele in northern China suggested the presence of selective pressures. Previous population genetic studies have suggested a role of natural selection in shaping the latitudinal inverse U-shaped gradient of C677T, which may involve factors such as regional climate, UV radiation, and folate intake (Pepe et al., 1998; Bauduer and Lacombe, 2005; Wang et al., 2012; Li et al., 2015). Compared with low-latitudes people living under strong UV, people living in mid-latitudes are less likely to undergo photolysis of *in vivo* folate. Compared with people living at high latitudes with sunlight deficiency and less vegetable foods, people living in mid-latitudes can improve their body folate status through dietary intake. Under these favorable conditions, the 677TT genotype can be preserved to the maximum extent. However, the exact mechanism underlying the adaption of an increased level of tHCY remains elusive.

The current study has several strengths. First, our study is the first ExWAS of tHCY in a well-sized Chinese population, based on which we could confirm the effects of known alleles identified in other populations and identify novel EAS-specific variants explaining additional tHCY variance. Second, we performed a replication study in an independent sample and fully adjusted potential confounders to ensure true positive findings. Third, we used an exome-wide conditional analysis method, and our results emphasized its efficiency in detecting the presence of multiple causal mutations. This study also has several limitations. The coverage of our exome-genotyping data is much lower than that whole genome sequencing data, so we could not examine the effects of some previously reported tHCY-associated SNPs in our study. The participants in our study were mostly from northern China (Beijing); without southern Chinese samples, we could not directly examine the relationship between the geographic distribution of 677T and HHCY incidence in China. Future studies with a higher geographical coverage together with the folate intake information would help to refine the hypothesis of geographic distribution of HHCY incidence in China.

In conclusion, the ExWAS of tHCY in a Chinese population detected the presence of multiple causal mutations at the *MTHFR* locus by identifying an EAS-specific missense variant C163T, which together with the well-known 677T allele affects tHCY in a compound heterozygote manner. The geospatial distribution of homozygote 677T genotype coincides with that of HHCY incidence in China, supplementing the current hypothesis about the high HHCY risk in northern China due to insufficient folate intake. A logistic model consisting of sex, age, and three DNA variants constructed in the discovery sample could fairly accurately predict the HHCY risk in the testing sample.

## Material and Methods

### Discovery Cohort

The establishment of the discovery cohort has been described in details previously (Fan et al., 2016). In brief, 9,540 participants aged ≥40 years were recruited from the Gucheng and Pingguoyuan communities of Shijingshan District in Beijing of China. Baseline survey was investigated from December 2011 to April 2012 as described previously (Fan et al., 2016). Among them, a total of 6,480 subjects were genotyped by ExomeChip. To obtain high-quality genotypes, strict criteria were applied to filter out low-quality genotypes. We undertook plate-, individual-, and variant-level checks to exclude poor-quality genotype calls from the dataset. A total of 5,959 samples passed the above quality control. We excluded 702 participants based on the identity by descent (IBD) analysis conducted in our samples. We used pi-hat ≥0.35 as cutoff to remove higher than the first degree (based on the pi-hat distribution, the unbiased estimate is 0.5) of close relatives by keeping the sample with the highest call rate for each family group. We then excluded 82 participants who did not have tHCY data. Finally, 5,175 eligible participants of Chinese Han ancestry were included in this analysis.

### Replication Sample

Two cohorts, myocardial infarction (MI) cohort and Chinese Academy of Sciences (CAS) cohort, were included in the replication stage. Both cohorts were from Beijing, China. Participants of MI cohort were hospitalized with MI in Department of Cardiology, Peking University First Hospital, from February 2005 to May 2013. Initially, a total of 593 participants had their genotypes tested using customized ExomeChip. The details of this cohort, methods, and primary results have been reported elsewhere. After data quality controls and exclusion of those without tHCY and other missing covariates, a total of 410 eligible Chinese Han ancestry MI patients were included in this analysis. The CAS participants consisted of 273 employees of CAS who were invited to join in a personalized health management during their annually physical examination. tHCY measurements and epidemiological information were collected before the health management period. After data quality controls, a total of 258 eligible Chinese Han ancestry participants were included in this analysis.

### Data Collection

Information, including demographic status, health behavior, disease history, and medical use, was collected using a standardized questionnaire in the discovery cohort and CAS cohort and extracted from in-hospital electronic medical records in the MI cohort.

Venous blood samples were obtained by venipuncture for all participants after overnight fasting. Plasma samples separated within 30 min and extracted DNA samples were stored at −80°C until measurement. For discovery sample, plasma tHCY was measured using an autobiochemical analyzer (AU480; Beckman Coulter, Brea, CA, United States) with the circulating enzymatic method in the core laboratory of the National Clinical Research Center for Kidney Disease of Nanfang Hospital in Guangzhou, China. The details of this method were subscribed in previous study (Momin et al., 2017). Serum creatinine (Scr) at baseline was measured on the Roche C8000 Automatic Analyzer (Roche Diagnostics, Basel, Switzerland) in the laboratory of the Chinese PLA General Hospital.

### Genotype and Quality Controls

All subjects were genotyped using the Asian ExomeChip, a specially designed exome array with a custom content of 58,317 variants on top of the standard Infinium HumanExome BeadChip (Illumina, San Diego, CA, United States), which integrated a total of 302,218 variants. Details of the Asian ExomeChip design has been described in previous studies (Zhang et al., 2014; Tang et al., 2015). In brief, the original design of the exome array includes 242,901 markers, with the majority of over 200K coding variants identified from ∼12,000 sequenced genomes and exomes of primarily European ancestry. The underrepresentation of non-European genomes in the original design limited the coverage of low-frequency variants in Asian populations. To allow the comprehensive genotyping across the full allele frequency spectrum, a custom panel of ∼30K nonsense/missense variants were added based on three independent Asian sequencing datasets of ∼1,000 Chinese samples.

A total of 5,959 individuals passed the individual-based QC criteria for filtering, which included a call rate of <99%, sex mismatch, and excess heterozygosity. We also performed variant-level quality control by excluding variants that have <99% genotype call rate or deviation from the Hardy–Weinberg equilibrium (p < 1E−4). In total, 282,456 variants passed the quality control. Among them, 119,020 variants were polymorphic variants in our data. Finally, after exclusion of related samples using a greedy algorithm (Sun et al., 2021) and variants with MAC ≤3, a total of 5,175 unrelated individuals with tHCY data and 89,131 autosomal variants were retained in further analysis.

### Statistical Analysis

The population structure analysis inferred by ADMIXTURE software (Alexander et al., 2009) of the discovery samples were conducted with CHB and CHS from the 1000 Genomes Project as reference. We ran ADMIXTURE using an LD pruned marker set of 25,121 variants, assuming 2 (K = 2) genetic clusters. To normalize the plasma tHCY, residuals were obtained using linear regression after adjusting for age, sex, smoking, drinking, creatinine, vitamin usage, and the first two principal components. Then inverse normal transformation residuals were created for analysis. The ExWAS of residuals of tHCY was carried out in PLINK v1.9 (Purcell et al., 2007) using linear regression model, assuming an additive allele effect. The genomic control inflation factor was close to 1.0 (*λ* = 1.03) in ExWAS and was not further considered. The *p*-values equal to or smaller than 5E−7 based on Bonferroni correction of 89,131 autosomal variants were considered as exome-wide significant. Conditional analysis of discovery ExWAS result was performed using PLINK v1.9 (Purcell et al., 2007). Results of discovery ExWAS and conditional analysis were visualized by Manhattan and Q–Q plots generated by an R package qqman (Turner, 2018). Regional LD analysis was conducted using a self-written R script LDplot using EAS data from the 1000 Genomes Project. Regional Manhattan plots were created by LocusZoom (Pruim et al., 2010) with LD population according to the 1000 Genomes Project ASN dataset. We conducted linear regression analysis using each identified variant as the explanatory variable, and the fitness *R*2 from this model was considered as the phenotype variance explained by the variants.

Identified variants were annotated using ANNOVAR (Version 2017-07–17) (Wang et al., 2010) with respect to the hg19 genome build. The allele frequencies of candidate variants in different populations were examined in the 2,504 subjects of the 1000 Genomes Project and visualized using R. The significant expression eQTL effects of identified SNP in all available tissues were annotated using the data from The Genotype-Tissue Expression project (GTEx, http://www.gtexportal.org/home/, data source: GTEx Analysis Release V8 (dbGaP Accession phs000424.v8.p2) (GTEx Consortium, 2013). The nominal *p*-value of each variant–gene pair was calculated from the genome-wide empirical *p*-value and the beta distribution model of each gene. The variant–gene pairs with a *p*-value lower than the gene-level threshold (0.05 false discovery rate) were considered significant enough to be included in the list of variant–gene pairs. All the variants with exome-wide significant association in the discovery analysis and conditional analysis were selected for replication with a focus on the top-associated SNP per region. The replication was carried out separately in a total of 668 samples, using linear regression adjusted for age, sex, smoking, and drinking, assuming an additive allele effect. For replication study, the significance level was set to 0.017 based on Bonferroni correction of three significant variants. Explained phenotypic variance was derived for identified independent associated variants using backward stepwise linear regression analyses. In order to check if the compound heterozygote, rather than double heterozygotes, may indeed explain the identified association, we inferred haplotypes using the expectation maximization algorithm implemented in R library haplo.stat (Schaid et al., 2002).

Fine-tuned individual-level data analyses were conducted in 5,175 unrelated Chinese individuals from discovery stage. HHCY status was defined with tHCY >15 μmol/L in both discovery samples and replication samples. A multiple linear regression analysis was conducted to access the independent effect of three variants. The model considers three identified variants in discovery analysis and conditional analysis (*MTHFR* C677T, *MTHFR* C136T and *DPEP1* rs1126464), together with sex and age as explanatory factors. We then applied multiple linear regression models including sex and age as predictors with or without the three variants developed in the discovery samples to the replication samples (N = 668) for tHCY level prediction. Prediction accuracy was estimated using R-squared correlation (*R*2) with tHCY phenotype. For HHCY risk prediction, multiple logistic regression models including sex and age as predictors with or without the three variants developed in the discovery samples were applied to the replication samples. Prediction accuracy was estimated using the AUC. AUC is the integral of ROC curves and ranges from 0.5 representing total lack of prediction (no better than flipping a coin) to 1.0 representing perfect prediction. Sensitivities and specificities were calculated using confusion matrices considering the predicted probability > *t* as the predicted shape type, where t optimized the sum of sensitivity and specificity.

## Data Availability

The datasets presented in this study can be found in online repositories. The names of the repository/repositories and accession number(s) can be found below: OMIX, China National Center for Bioinformation / Beijing Institute of Genomics, Chinese Academy of Sciences (https://ngdc.cncb.ac.cn/omix), accession OMIX499.
