# Supplementary material for: Exome-Wide Association Study Identifies East Asian-Specific Missense Variant MTHFR C136T Influencing Homocysteine Levels in Chinese Populations RH: ExWAS of tHCY in a Chinese Population
Source: Front Genet. 2021 Oct 11;12:717621. doi: 10.3389/fgene.2021.717621 (PMC8542906; doi:10.3389/fgene.2021.717621)
Supplement: Supplementary file 1 [file Image5.PDF]

a *CDK10*

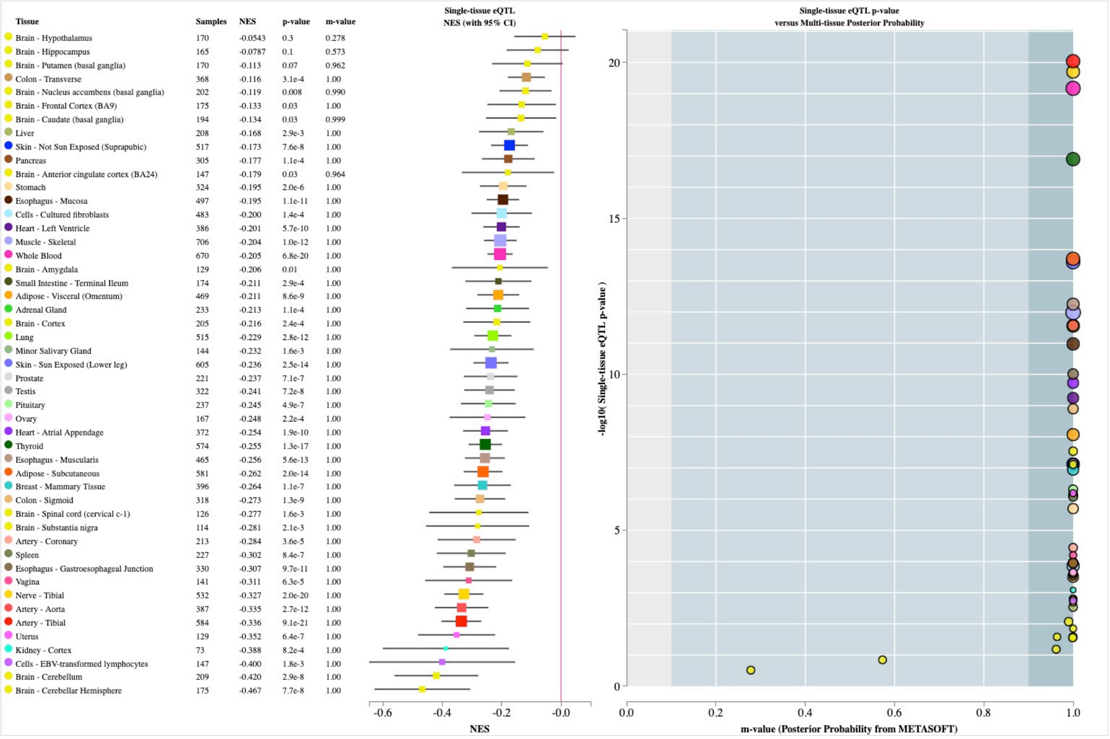

b *CHMP1A*

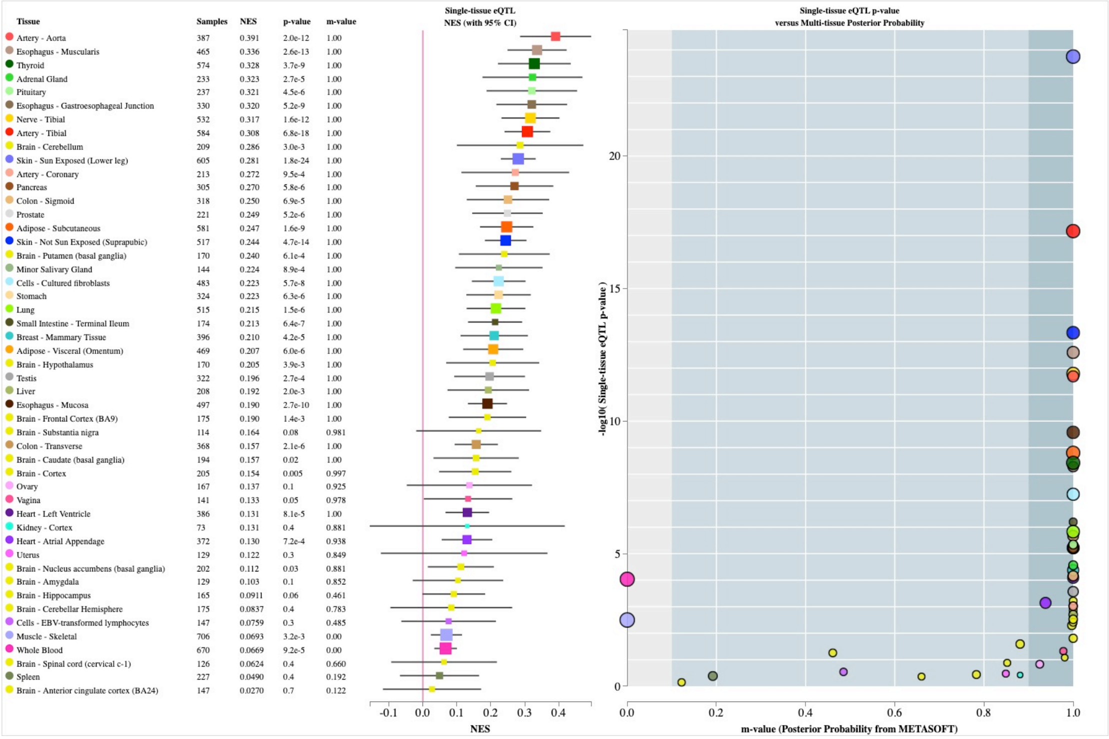

c *FANCA*

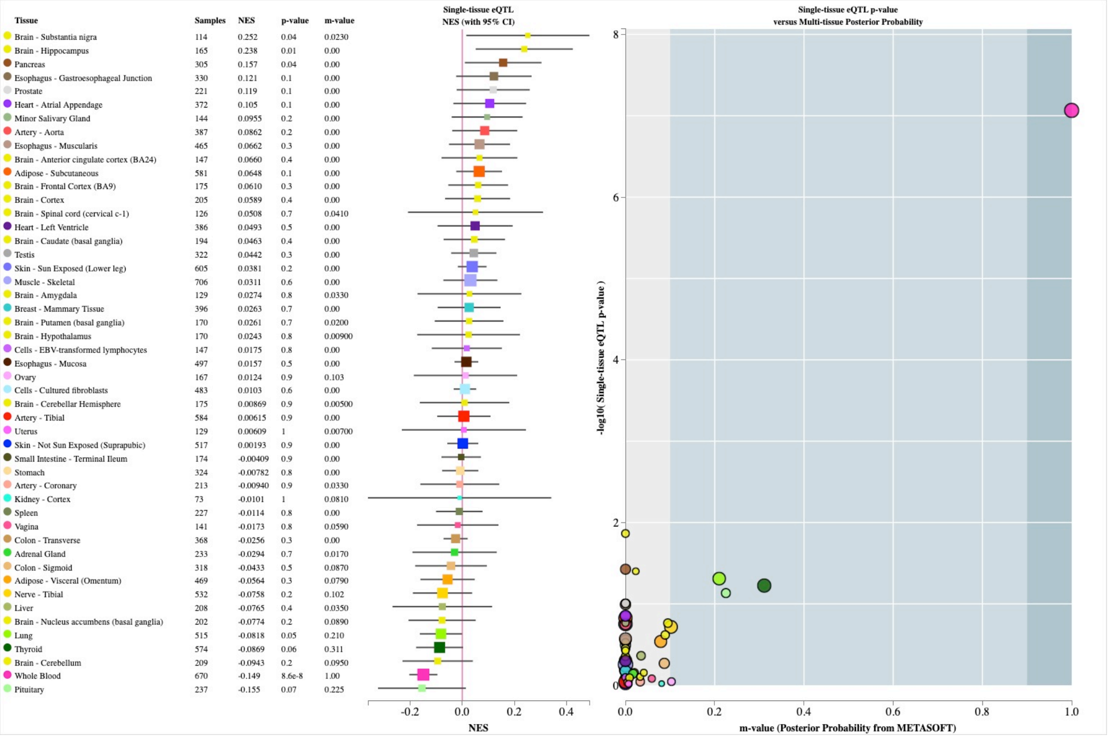

d *VPS9D1*

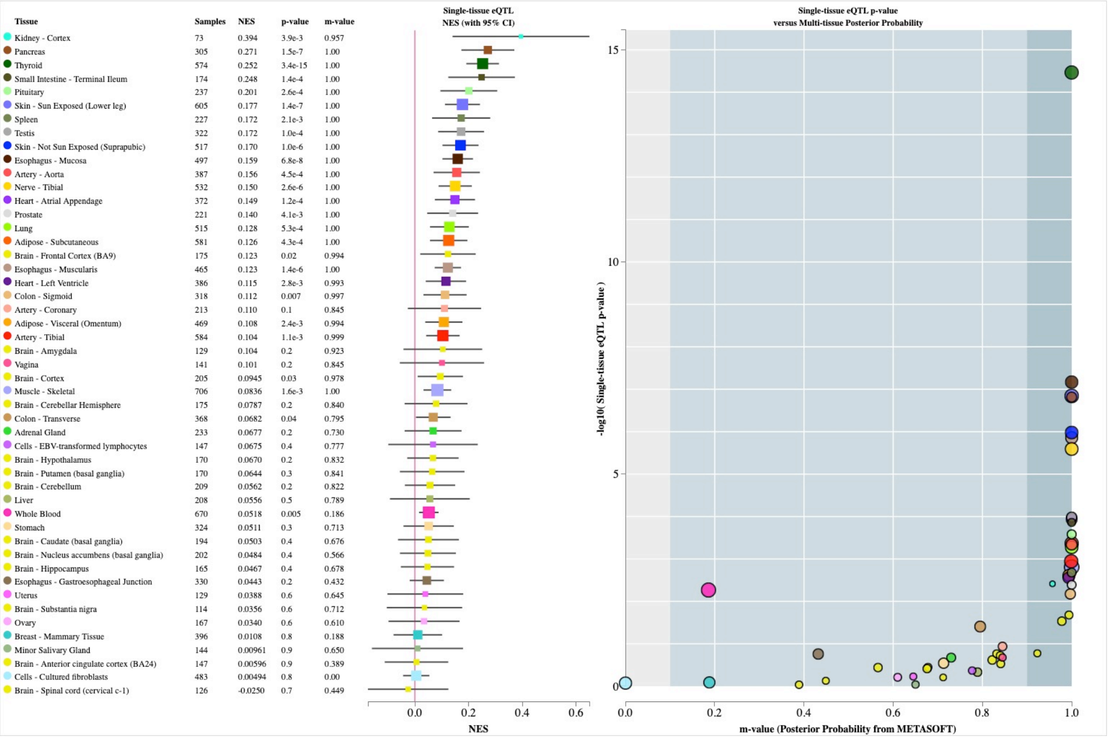

Figure S5. (a) The multi-tissue eQTL comparison of rs1126464 (*DPEP1*) and *CDK10*. (b) The multi-tissue eQTL comparison of rs1126464 and *CHMP1A*. (c) The multi-tissue eQTL comparison of rs1126464 and *FANCA*. (d) The multi-tissue eQTL comparison of rs1126464 and *VPS9D1*.
