# Supplementary material for: Exome-Wide Association Study Identifies East Asian-Specific Missense Variant MTHFR C136T Influencing Homocysteine Levels in Chinese Populations RH: ExWAS of tHCY in a Chinese Population
Source: Front Genet. 2021 Oct 11;12:717621. doi: 10.3389/fgene.2021.717621 (PMC8542906; doi:10.3389/fgene.2021.717621)
Supplement: Supplementary file 3 [file Image4.pdf]

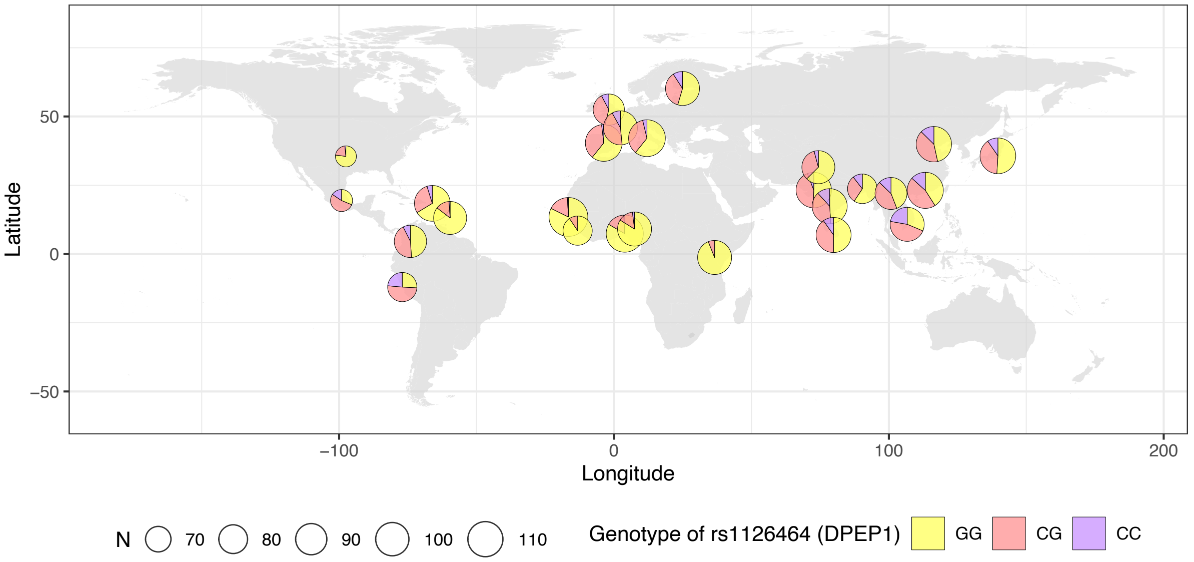

Figure S4. Worldwide prevalence patterns for genotypes of rs1126464 (*DPEP1*). Map displaying the geospatial distribution of the prevalence patterns for genotypes of rs1126464 across the world. The map was drawn based on the genotypes of 2,504 subjects obtained from 1000 Genome project datasets for this SNP. The pie denotes the sampling locations.
