# Supplementary material for: Exome-Wide Association Study Identifies East Asian-Specific Missense Variant MTHFR C136T Influencing Homocysteine Levels in Chinese Populations RH: ExWAS of tHCY in a Chinese Population
Source: Front Genet. 2021 Oct 11;12:717621. doi: 10.3389/fgene.2021.717621 (PMC8542906; doi:10.3389/fgene.2021.717621)
Supplement: Supplementary file 4 [file Image2.PDF]

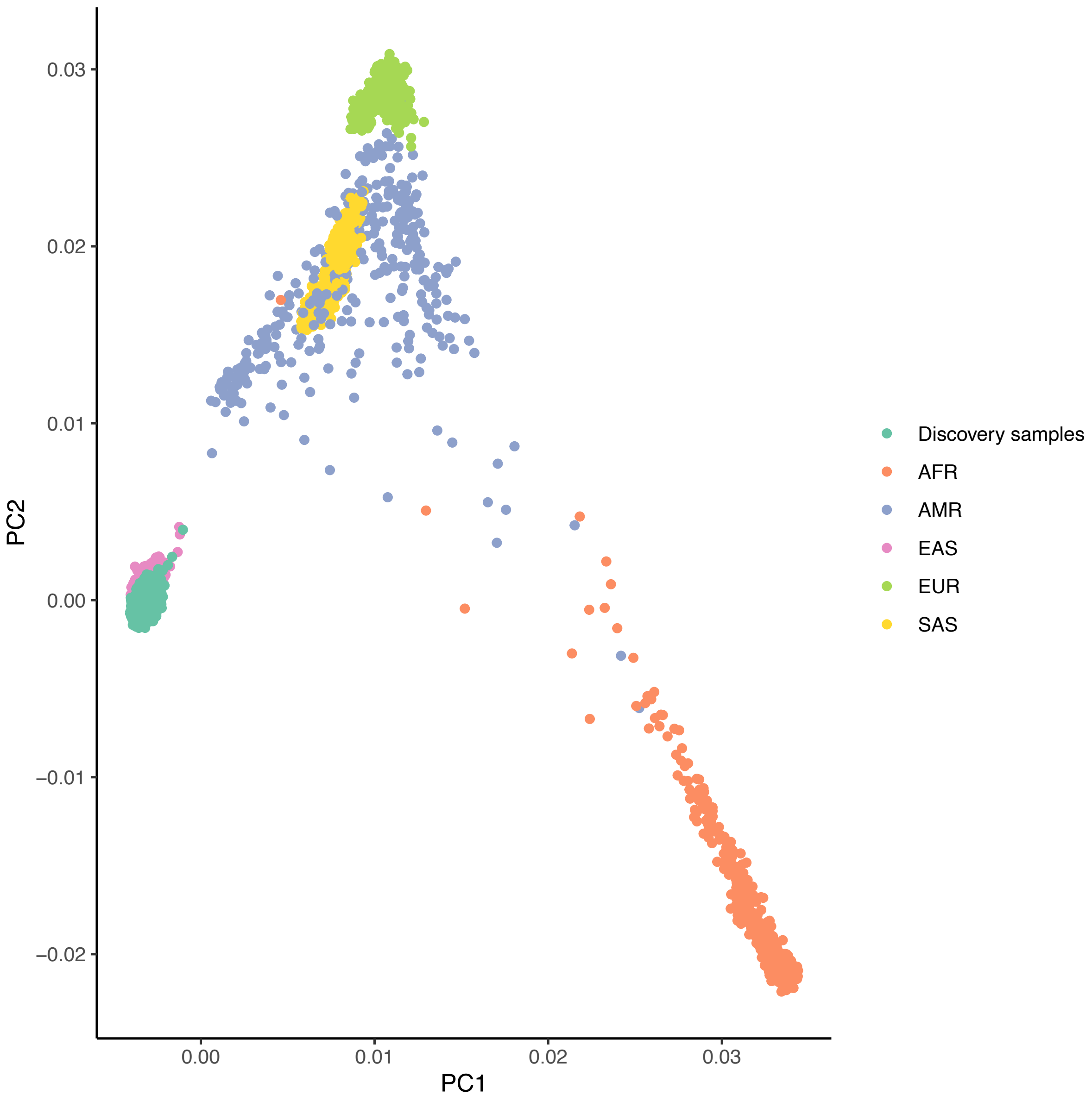


**Figure S2.** Principal component analysis plot of the discovery sample and 1000 Genomes Phase III samples. The colors of the points represent five populations from 1000 Genome and discovery samples in this study.
