# Supplementary material for: Exome-Wide Association Study Identifies East Asian-Specific Missense Variant MTHFR C136T Influencing Homocysteine Levels in Chinese Populations RH: ExWAS of tHCY in a Chinese Population
Source: Front Genet. 2021 Oct 11;12:717621. doi: 10.3389/fgene.2021.717621 (PMC8542906; doi:10.3389/fgene.2021.717621)
Supplement: Supplementary file 5 [file Image3.PDF]

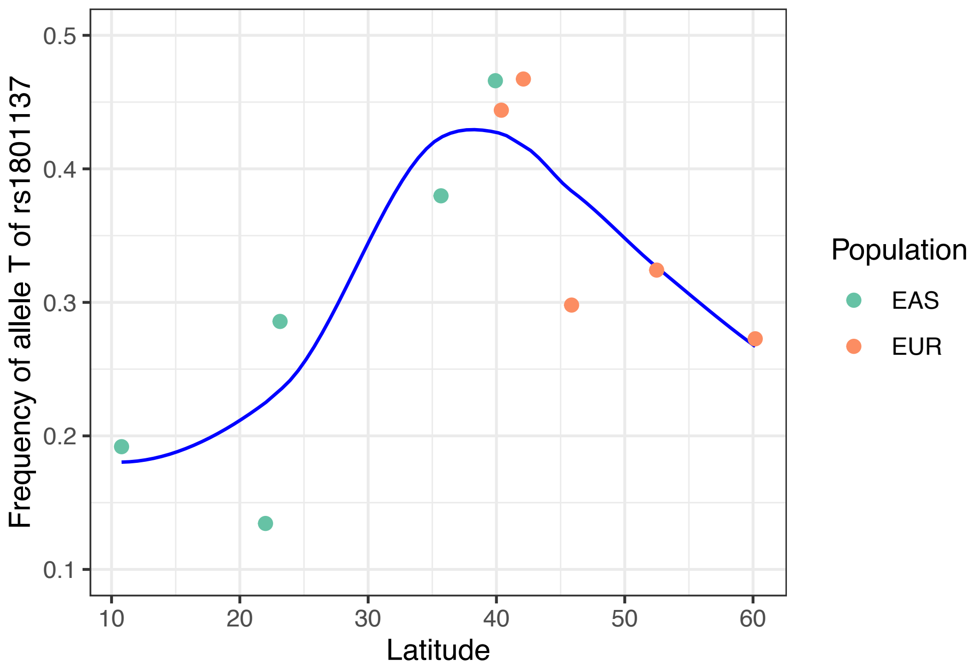

Figure S3. The allele frequency of *MTHFR* C677T (rs1801133) based on the genotypes of East Asians and Europeans obtained from 1000 Genomes Project datasets and the corresponding latitude of each population.
