# Supplementary material for: Exome-Wide Association Study Identifies East Asian-Specific Missense Variant MTHFR C136T Influencing Homocysteine Levels in Chinese Populations RH: ExWAS of tHCY in a Chinese Population
Source: Front Genet. 2021 Oct 11;12:717621. doi: 10.3389/fgene.2021.717621 (PMC8542906; doi:10.3389/fgene.2021.717621)
Supplement: Supplementary file 8 [file Image1.PDF]

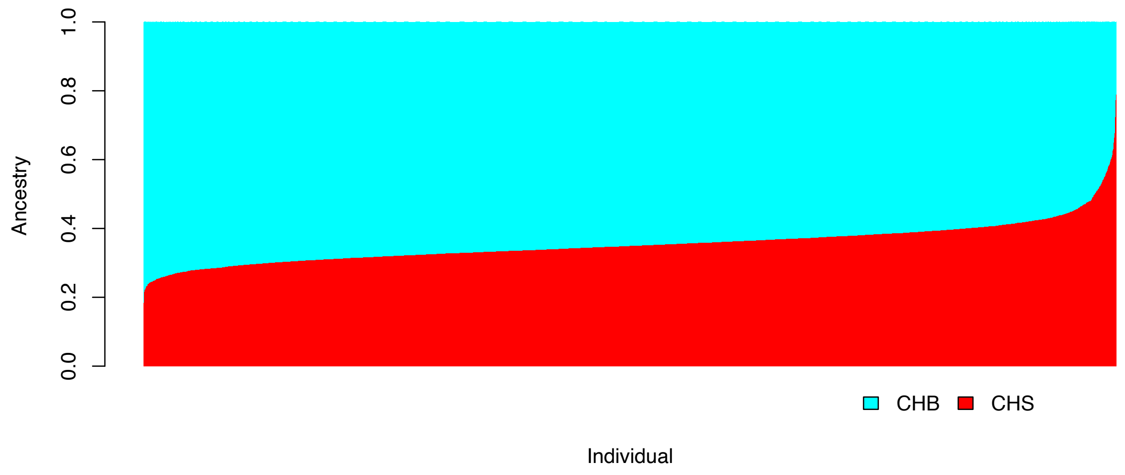

Figure S1. Population structure inferred using ADMIXTURE analysis with northern (CHB) and southern (CHS) Chinese from 1000 Genome Projects as reference. Each individual is represented by a vertical (100%) stacked column indicating the proportions of ancestry in 2 constructed ancestral populations.
